# Construction of a risk assessment model of cardiovascular disease in a rural Chinese hypertensive population based on lasso‐Cox analysis

**DOI:** 10.1111/jch.14403

**Published:** 2021-12-09

**Authors:** Nanxiang Ouyang, Guangxiao Li, Chang Wang, Yingxian Sun

**Affiliations:** ^1^ Department of Cardiology First Hospital of China Medical University Shenyang China; ^2^ Department of Medical Record Management First Hospital of China Medical University Shenyang China

**Keywords:** cardiovascular disease, hypertension, risk assessment model

## Abstract

Many assessments have been used to predict cardiovascular risks in the general population, but their applicability in patients with hypertension needs to be further evaluated. In the current study, a cardiovascular risk assessment model was constructed in a hypertensive population. This prospective cohort study was conducted with cardiovascular examinations in rural northeast China in 2012 and 2013, and followed up to collect cardiovascular events in 2015 and 2018. Data were derived from 4763 hypertensive patients who were free of cardiovascular disease (CVD) at baseline and completed follow‐up. After lasso regression was used to screen for risk factors of CVD at baseline, a multivariate Cox regression risk model was established and a nomogram was developed. The model was validated using an independent test set (one third of data not used for model building). Among 4763 patients, 354 (7.43%) had a cardiovascular event during a median follow‐up of 4.66 years. Nine risk factors were screened by lasso regression, including sex, age, current smoking, body mass index (BMI), history of transient ischemic attack (TIA), family history of hypertension, family history of stroke, physical labor intensity, and high low‐density lipoprotein cholesterol (LDL‐C). The c‐index of the CVD model was 0.707, and that of an updated model with baseline blood pressure was 0.732. In the validated cohort the respective c‐indexes were 0.665 and 0.714. An assessment model of CVD risk was established in a hypertensive population which may provide an original prevention strategy for hypertensive populations in rural China, and further reduce the CVD burden.

## 1 INTRODUCTION

Cardiovascular disease (CVD) is the leading cause of death in China and worldwide.^1^, ^2^ With the acceleration of population aging and urbanization in China the increasing prevalence of CVD risk factors is obvious, leading to a continuous increase in the number of CVDs. At present CVDs are the leading cause of death in urban and rural residents in China, and the death rate is far higher than that of tumors and other diseases. The mortality rate due to CVDs in rural residents has increased significantly and continues to be higher than that of urban residents.^3^, ^4^ From 1980 to 2016, the number of discharged patients with CVD and the cost of hospitalization continued to rise in China.^4^ The high morbidity and mortality of CVD have imposed a heavy economic burden on China. Therefore, CVD prevention and control in rural areas of China is highly desirable.

Hypertension has been recognized as an independent risk factor for cardiovascular events.^5^ The risk of cardiovascular events in hypertensive patients is 2–5 times higher than that in normotensive patients.^6^ More than half of CVD cases in China are related to hypertension.^7^ Hypertension is a major cause of CVD and an important cause of premature death in China.^2^ The latest national data show that the Chinese hypertensive population has exceeded 300 million.^8^ The prevalence of hypertension in rural areas of northeast China is as high as 50%.^9^ Therefore, increased prevention and control of cardiovascular events in Chinese rural hypertensive populations is required, and overall cardiovascular risk assessment and risk stratification are important strategies for the prevention and treatment of CVDs.

## 2 METHODS

### 2.1 Study population

The Northeast China Rural Cardiovascular Health Study was a prospective cohort investigation conducted from 2012 to 2018 in rural areas of northeastern China. Dawa, Zhangwu, and Liaoyang counties in Liaoning Province were selected using a multistage, randomly stratified cluster‐sampling scheme. A total of 11,956 participants aged ≥ 35 years were enrolled at baseline in 2012 and 2013. All study patients were invited to return for follow‐up visits during both 2015 and 2017 or 2018. A total of 1607 individuals refused or were lost to follow‐up, and 10,349 participants agreed and attended at least one follow‐up visit. In the present analysis participants with normal blood pressure (*n* = 4976) or with CVD (*n* = 610) were excluded, leaving 4763 participants with hypertension who were free of CVD at baseline. The data were randomly divided into a training set (*n* = 3176, two thirds of the data) for model construction and an independent test set (*n* = 1587, one third of the data) for model validation. Figure S1 shows the patient sample size and exclusion criteria. The Ethics Committee of China Medical University (Shenyang, China) approved the research protocol, and written informed consent was formally obtained from all participants.

### 2.2 Study variables

Physical examinations and detailed methodology on the data collection process have been described elsewhere.^9^, ^10^ Detailed information on demographic characteristics, socioeconomic information, dietary and lifestyle factors, medical history, and medication history (self‐reported use of antihypertensive, lipid‐lowering, or hypoglycemic drugs) were obtained via interviews using a standardized questionnaire. Three blood pressure (BP) measurements were assessed after a 5‐minute rest in a seated position using a standardized automatic electronic sphygmomanometer (HEM‐907; Omron, Tokyo, Japan). The mean of the three measurements was used in the analysis. Body weight and height were measured while the participants were wearing light indoor clothes without shoes, and body mass index (BMI) was calculated. Fasting blood samples were obtained after fasting for at least 12 hours to measure serum glucose, lipids levels, serum creatinine, blood electrolytes, and other routine blood biochemical indexes (Olympus AU 640, Tokyo, Japan).

### 2.3 Definitions

Hypertension was defined as systolic BP (SBP) ≥ 140 mm Hg and/or diastolic BP (DBP) ≥ 90 mm Hg and/or the administration of antihypertension drugs within 2 weeks.^11^ Diabetes mellitus was defined as having a fasting glucose level ≥ 7.0 mmol/L and/or a self‐reported diagnosis that was previously determined by a physician.^12^ Physical labor intensity was defined as occupational physical activity intensity and categorized into three groups(light; moderate; heavy), which has been presented elsewhere.^9^ Current smoking was defined as self‐reported at least one cigarette/day.^13^ Current drinking was defined as any dose of alcohol at least one time/week as reported by the patient.^14^ Transient ischemic attack (TIA) history, stroke history, and coronary heart disease (CHD) history were established via a combination of self‐reported and medical records. High low‐density lipoprotein cholesterol (LDL‐C) was defined as ≥ 3.4 mmol/L (130 mg/dL).^15^

### 2.4 Adjudication of outcomes

The primary outcome in the current study was incident CVD. The median follow‐up period was 4.66 years. CVD was defined as fatal or nonfatal CHD and stroke.^10^ Adjudication of the occurrence of CHD and stroke has been described previously.^10^ CHD was defined as hospital‐diagnosed angina and myocardial infarction, CHD death, or any revascularization procedure. Stroke was defined as a new neurologic deficit that was not attributable to apparent nonvascular causes, lasting more than 24 h. TIA was excluded. For deceased participants, death certificates that included cause of death were obtained from the Chinese Centers for Disease Control and Prevention. All possible CHD and stroke events were adjudicated by the End point Assessment Committee.

### 2.5 Statistical analysis

When continuous variables conformed to a normal distribution the mean and standard deviation were evaluated, and Student's *t*‐test or one‐way analysis of variance were used for statistical analysis. When the variable type is classified variable the corresponding constituent ratio of each category was calculated, and chi square analysis or Fisher's exact test were used for statistical analysis.

Candidate variables were screened via lasso regression to achieve dimensionality reduction and optimization of the model, and prevent the over‐fitting phenomenon. All variables selected were included to build a nomogram to investigate the probability of incident CVD in each individual in the next 2 or 4 years.

Bootstrapping with 1000 resamples was used for internal validation of the nomogram model. The indicators of internal validation included c‐index and calibration, which respectively, represented the prediction accuracy and prediction compliance of the nomogram prediction model. Calibration degree was represented by a calibration graph. The area under the receiver operating characteristic (ROC) curve was calculated as a supplementary index of prediction accuracy.

The glmnet package of R was used for variable selection in the lasso method, and the rms package of R was used for drawing and internal verification of the nomogram (c‐index and calibration chart). ROC curves were drawn with the survival ROC package.

Comparisons of the performance among the nomograms were conducted by calculating the category‐free net reclassification improvement (NRI)^16^ and integrated discrimination improvement (IDI). The Z test was used to calculate *p* values of IDI. Cox proportional hazards regression modeling was conducted using the survival package. The main statistical analysis software used in this study was R version 3.5.1, and two‐tailed *p* values < .05 were considered statistically significant.

## 3 RESULTS

### 3.1 Baseline characteristics

Baseline characteristics of the participants in the development cohort and the validation cohort are shown in Table 1. The mean age of the development cohort was 56.69 ± 10.22 years, and that of the validation cohort was 56.00 ± 10.02 years (*p*  =  .03). However, there was no significant differences in the proportion of age stratification between the two cohorts (*p* = .07). The development cohort included 1587 men (50.0%) and 1589 women (50.0%), and the validation cohort included 797 men (50.2%) and 790 women (49.8%) (*p* = .87). The mean SBP of the development cohort was 158.35 ± 18.92 mm Hg, and that of the validation cohort was 157.95 ± 19.32 mm Hg (*p* = .50). The mean DBP of the development cohort was 88.81 ± 11.08 mm Hg, and that of the validation cohort was 88.93 ± 11.11 mm Hg (*p* = .72). There were no significant differences in any baseline characteristics (including current smoking, current drinking, body mass index, and family history) between the two cohorts.

**TABLE 1:** Comparison of basic characteristics between development cohort and validation cohort

Basic characteristics	Development cohort No. (%)	Validation cohort No. (%)	*p*
Number of patients	3176	1587	
Age, year (SD)	56.69 ± 10.22	56.00 ± 10.02	.03
35∼44	445 (14.0)	247 (15.6)	.07
45∼54	937 (29.5)	480 (30.2)	
55∼64	1141 (35.9)	582 (36.7)	
65∼	653 (20.6)	278 (17.5)	
Sex			0.87
Male	1587 (50.0)	797 (50.2)	
Female	1589 (50.0)	790 (49.8)	
Current smoking	1118 (35.2)	595 (37.5)	.12
Current drinking	833 (26.2)	423 (26.7)	.75
BMI, kg/m^2^(SD)	25.48 ± 3.56	25.58 ± 3.61	.36
Mean SBP	158.35 ± 18.92	157.95 ± 19.32	.50
Mean DBP	88.81 ± 11.08	88.93 ± 11.11	.72
Education			.44
Illiterate	353 (11.1)	157 (9.9)	
Primary school	1391 (43.8)	693 (43.7)	
Junior high school	1158 (36.5)	584 (36.8)	
High school and above	274 (8.6)	153 (9.6)	
Diabetes	472 (14.9)	214 (13.5)	.18
TIA	101 (3.2)	53 (3.3)	.77
Family history of hypertension	821 (25.9)	389 (24.5)	.32
Family history of stroke	553 (17.4)	299 (18.8)	.23

Values are shown as *n* (%).

*Abbreviations*: BMI, body mass index; TIA, transient ischemic attack; SBP, systolic blood pressure; DBP, diastolic blood pressure.

### 3.2 Variable selection

Lasso‐penalized Cox analysis was performed in the development cohort to narrow the candidate independent variables. Because all participants were hypertensive patients, different levels of BP were not screened. The incorporation of BP levels into the updated model was used to highlight the importance of blood pressure control level on the CVD outcomes. Different BP levels correspond to different risk levels of CVD,^17^, ^18^ therefore baseline SBP and DPB levels were used for subsequent updating of the model. The process of independent variable selection via lasso regression is shown in Figure S2. Nine variables were selected: sex, age, current smoking, BMI, history of TIA, family history of hypertension, family history of stroke, physical labor intensity, and high LDL‐C. In multivariate Cox regression analysis these nine variables were risk factors for CVD in patients with hypertension in the development cohort (Table S1).

### 3.3 Constructing and validating a predictive nomogram (model A)

**FIGURE 1:**
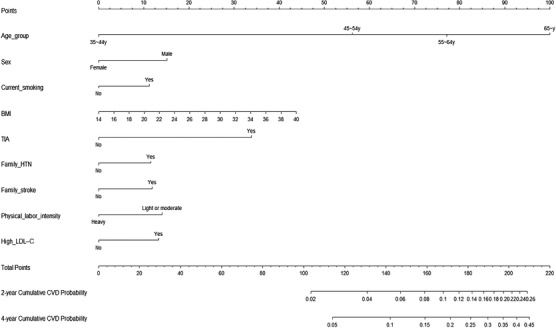
Model A: Nomogram for predicting 2‐year and 4‐year cumulative incidence of CVD in a hypertensive population

**FIGURE 2:**
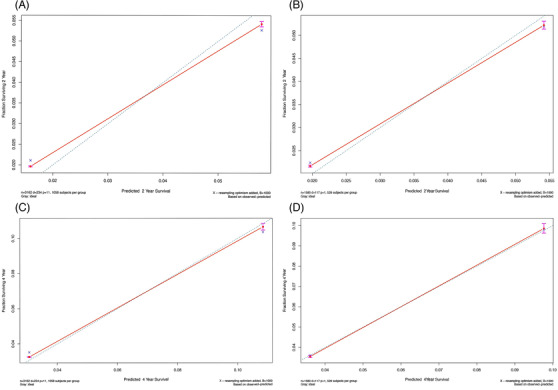
Calibration charts. (A, B) Calibration chart of 2‐year cumulative CVD risk prediction in the development cohort and the validation cohort (model A). (C, D) Calibration chart of 4‐year cumulative CVD risk prediction in the development cohort and the validation cohort (model A)

A nomogram was constructed to predict 2‐year and 4‐year CVD incidence in 3176 participants with hypertension using the above‐described nine independent prognostic factors (Figure 1). The c‐indexes were 0.707 (95% confidence interval [CI] 0.675–0.739) for the development cohort and 0.665 (95% CI 0.621–0.709) for the validation cohort. The areas under the curve (AUCs) of the 2‐year cumulative CVD incidence were 0.697 in the development cohort and 0.658 in the validation cohort (Figure S3A, S3B). The AUCs of the 4‐year cumulative CVD incidence were 0.718 in the development cohort and 0.682 in the validation cohort (Figure S3C, S3D). The predicted and actual values of cumulative CVD in the development cohort and validation cohort were in good agreement with each other after 2 and 4 years of follow‐up (Figure 2).

### 3.4 Updating and reevaluation of the model (model B)

**FIGURE 3:**
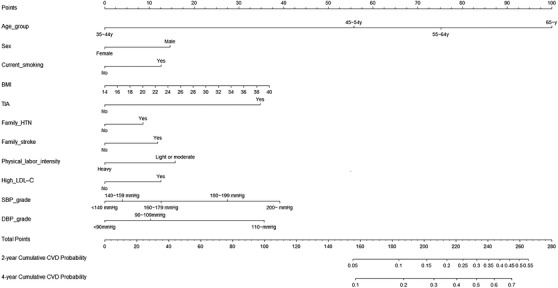
Model B: Nomogram for predicting 2‐year and 4‐year cumulative incidence of CVD in a hypertensive population

**FIGURE 4:**
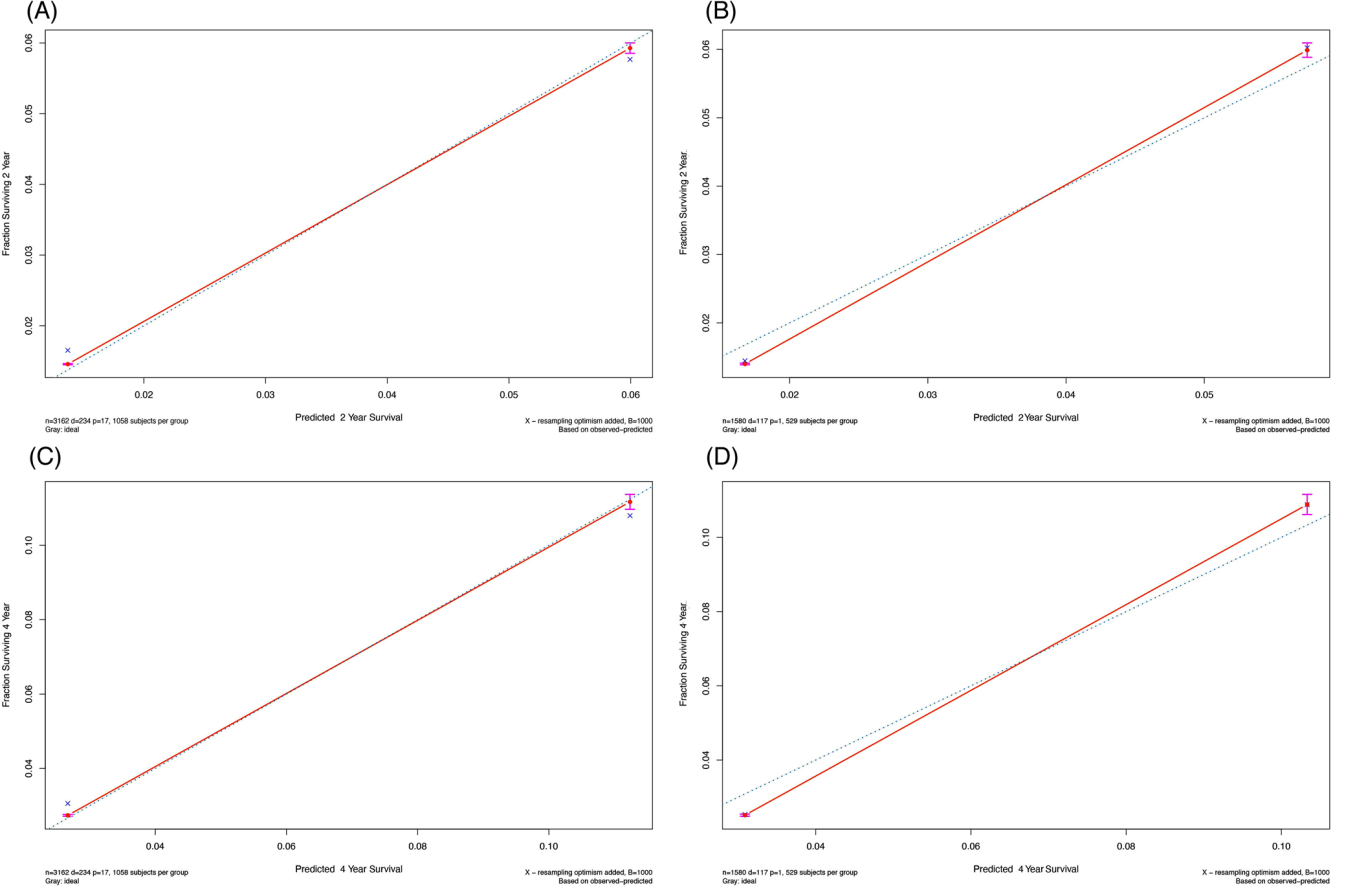
Calibration charts. (A, B) Calibration chart of 2‐year cumulative CVD risk prediction in the development cohort and the validation cohort (model B). (C, D) Calibration chart of 4‐year cumulative CVD risk prediction in the development cohort and the validation cohort (model B)

In univariate Cox regression mean SBP and mean DBP were risk factors for CVD, with respective hazard ratios of 1.024 and 1.019 (*p* < .001; Table S2). Model A was updated with SBP and DBP grading levels. The multivariate Cox regression model after the incorporation of updated variables is shown in Table S3. The updated predictive nomogram is shown in Figure 3, and the calibration plots are shown in Figure 4. In the updated model the c‐index for the development cohort was 0.732 (95% CI 0.702–0.762), and that for the validation cohort was 0.714 (95% CI 0.672–0.756). The AUCs of the 2‐year cumulative CVD incidence were 0.737 in the development cohort and 0.726 in the validation cohort (Figure S4A, S4B). The AUCs of the 4‐year cumulative CVD incidence were 0.745 in the development cohort and 0.727 in the validation cohort (Figure S4C, S4D). The calibration and c‐index of model B were better than those of model A.

### 3.5 Indicators for comparison between the original model A and the updated model B: NRI and IDI

NRI was defined as the difference in the proportions of participants with events correctly assigned a higher probability (NRI^+^) and participants without events correctly assigned a low probability (NRI^–^) by an updated model compared with the old model.^19^, ^20^ The total NRI was statistically significant with respect to both the 2‐year cumulative incidence of CVD and the 4‐year cumulative incidence of CVD (Table 2), which showed that model B was better than model A. NRI^–^ was statistically significant, which indicated that the prediction accuracy of model B for individuals without events was better than that of model A. In addition, the reclassification results of the updated model compared with the primary model indicated that the IDI index of the development cohort and the validation cohort were both greater than 0 for 2‐year cumulative CVD incidence and 4‐year cumulative CVD incidence (all with *p *< .05). This indicated that the reclassification of the updated model after the addition of SBP and DBP was more accurate and the model performed better.

**TABLE 2:** Indicators of comparison between the model A and the model B (NRI, IDI)

Index	2‐year cumulative CVD incidence		4‐year cumulative CVD incidence	
	Development cohort	Validation cohort	Development cohort	Validation cohort
NRI (95%CI)	0.473 (0.307, 0.682)	0.448 (0.166, 0.757)	0.376 (0.245, 0.524)	0.318 (0.098, 0.514)
NRI^+^ (95%CI)	0.018 (‐0.148, 0.232)	0 (‐0.280, 0.311)	−0.079 (‐0.213, 0.062)	−0.126 (‐0.344, 0.075)
NRI^‐^ (95%CI)	0.454 (0.426, 0.487)	0.448 (0.403, 0.492)	0.455 (0.426, 0.484)	0.444 (0.398, 0.483)
IDI (*P* value)	0.013 (*P *< 0.001)	0.008 (0.01)	0.022 (*P *< 0.001)	0.015 (*P *< 0.01)

*Abbreviations*: NRI, Net Reclassification Improvement; IDI, Integrated Discrimination Improvement.

## 4 DISCUSSION

Since the development of Kannel and coworkers^21^ pioneering Framingham CVD prediction model in the 1970s there has been great progress in CVD risk assessment. Chinese scholars have established CHD prediction models^22^ and a 10‐year risk prediction model of ischemic CVD,^23^ but these models were derived from risk factors more than 10 years ago. In 2016, Gu and coworkers^24^ established the China‐PAR model, but it was based on the general population. Our prediction model of CVD risk was established based on hypertensive patients in rural communities in China.

Since the publication of SPRINT research results,^25^ discussion about reducing the target value of BP control to achieve greater clinical benefits in patients with CVD has lasted for 5 years. The BPLTCC study^26^ published at the 2020 ESC conference further confirmed that the relative risk of major cardiovascular events decreased by approximately 10% for every 5‐mm Hg decrease in SBP. The present study indicates that the better BP control is, the lower the risk of CVD is, which is in accordance with previous researches. Our model was established, validated, and updated in strict accordance with the Transparent Reporting of a Multivariable Prediction Model for Individual Prognosis or Diagnosis statement.^27^ NRI and IDI indicated that the predictive capacity of the updated model was better than that of the original model. Briefly, the calibration and discrimination of the model were fully evaluated, and it can be used to predict CVD in rural hypertensive populations in China, and provide individualized strategies for early prevention and treatment of hypertensive populations.

Compared with the China‐PAR model, our model added risk factors for common CVDs such as SBP level, DBP level, family histories, history of TIA, and BMI, and physical labor intensity and it also used high LDL‐C, and other traditional cardiovascular risk factors to assess the risk of CVD. With regard to family history, the highest score in the nomogram model was 32. After family history was applied to the previous prediction models, the calibration degrees of prediction were good.^24^, ^28^, ^29^ This is consistent with the current study. Obesity has been an established risk factor for increased cardiovascular disease.^30^ The obesity paradox does exist in patients with hypertension, that is, the prevalence of adverse events in thin or normal weight patients with hypertension are higher than those of obese patients.^31^, ^32^ Our results as well as established studies^33^, ^34^, ^35^ are inconsistent with the obesity paradox. This may be due to age factors. Among the elderly ≥ 60 years old, overweight and obesity are potential factors to reduce the risk of cardiovascular death.^36^ The average age of our development cohort was 56.69 ± 10.22 years, less than 60 years old. Our study indicated that with the increasing of BMI, the cardiovascular risks of hypertensive patients also increased. Many professional organizations around the world have shown that exercise can reduce blood pressure.^37^ A meta‐analysis in 2019 clearly showed that there was an opposite dose‐response relationship between physical activity and cardiovascular mortality in adults with hypertension,^38^ which is consistent with our results. There is a study demonstrated that the incidence of CVD was positively correlated with LDL‐C concentrations.^39^ Similarity, this study also supports the view.

The current study had several limitations. The number of people used to derive the model was not large enough, and the number of cardiovascular events is only 237 in the development cohort and 117 in the validation cohort, which may limit conclusions that can be drawn from the results. Although the model was validated by 1/3 of the random samples with different baseline characteristics, it was not validated in other populations. A small amount (statistically acceptable) of the baseline data was lost. Although multiple imputation was used, these lost data may limit interpretation of the results. Lastly, the defect of the definition of CVD does not include incident heart failure may limit our results.

In conclusions, in the current study a new prognostic model and nomogram to predict the short‐term risk of CVD (2‐year and 4‐year) in hypertensive populations were established based on a combination of traditional cardiovascular risk factors and clinical biochemical indexes. The model can identify risk individuals among hypertensive patients in rural areas of China, and facilitate individual clinical decision‐making for prevention and treatment.

## AUTHORS CONTRIBUTION

Yingxian Sun and Nanxiang Ouyang contributed to the conception or design of the work. Guangxiao Li and Chang Wang contributed to the acquisition, analysis, or interpretation of data for the work. Nanxiang Ouyang drafted the manuscript. All gave final approval and agree to be accountable for all aspects of work ensuring integrity and accuracy.

## CONFLICTS OF INTEREST

There are no conflicts of interest.

## Supporting information

Supporting material
