# Supplementary material for: Construction of a risk assessment model of cardiovascular disease in a rural Chinese hypertensive population based on lasso‐Cox analysis
Source: J Clin Hypertens (Greenwich). 2021 Dec 9;24(1):38–46. doi: 10.1111/jch.14403 (PMC8783342; doi:10.1111/jch.14403)

**Supplemental material**

**eTable1**.Univariate Cox regression analysis for the risk of CVD in the development cohort

**eTable2.**Univariate Cox regression analysis of mean SBP and mean DBP on the risk of CVD

**eTable3.**Cox proportional hazards model parameters of CVD (Update model)

**eFigure1.**Flow chart of participant recruitment and derivation of the population used in the final analysis.

**eFigure2.** The process of independent variable selection by lasso regression. (A) Penalty process of influencing factors in the lasso model. (B) Change of the best penalty coefficient (λ: lambda) in the lasso regression model.

**eFigure3.** ROC curves of the model. (A, B) ROC curves of 2-year cumulative incidence of CVD in the development cohort and the validation cohort (model A). (C, D) ROC curves of 4-year cumulative incidence of CVD in the development cohort and the validation cohort (model A).

**eFigure4.** ROC curves of the model. (A, B) ROC curves of 2-year cumulative incidence of CVD in the development cohort and the validation cohort (model B). (C, D) ROC curves of 4-year cumulative incidence of CVD in the development cohort and the validation cohort (model B).

**eTable1.** Multivariate risk Cox regression model based on independent variables screened by lasso method

| Variables | Coefficient | HR | 95%CI | *P* |
| --- | --- | --- | --- | --- |
| Age group, yrs |  |  |  |  |
| 35~44 | Reference | 1.00 | - | *-* |
| 45~54 | 1.44 | 4.22 | 1.67-10.66 | <0.01 |
| 55~64 | 1.97 | 7.20 | 2.91-17.72 | <0.001 |
| 65~ | 2.56 | 12.91 | 5.17-32.23 | <0.001 |
| Male *vs.* Female | 0.39 | 1.45 | 1.12-1.96 | <0.01 |
| Current smoking | 0.29 | 1.33 | 1.01-1.76 | 0.04 |
| BMI | 0.04 | 1.04 | 1.01-1.08 | 0.03 |
| TIA | 0.87 | 2.38 | 1.50-3.78 | <0.001 |
| High LDL-C | 0.34 | 1.41 | 1.07-1.84 | 0.01 |
| Family of Hypertension | 0.30 | 1.35 | 1.00-1.81 | 0.04 |
| Family of stroke | 0.30 | 1.35 | 0.98-1.87 | 0.06 |
| Physical labor intensity |  |  |  |  |
| Light or moderate | Reference | 1.00 | - | *-* |
| Heavy | -0.36 | 0.70 | 0.51-0.95 | 0.02 |

BMI, body mass index; TIA, transient ischemic attack; LDL-C, low-density lipoprotein cholesterol.

**eTable2.**Univariate Cox regression analysis of mean SBP and mean DBP on the risk of CVD

| Variables | Coefficient | HR | 95%CI | *P* |
| --- | --- | --- | --- | --- |
| Mean SBP | 0.024 | 1.024 | 1.018-1.030 | <0.001 |
| Mean DBP | 0.019 | 1.019 | 1.008-1.031 | 0.001 |

SBP, systolic blood pressure; DBP, diastolic blood pressure

**eTable3.**Cox proportional hazards model parameters of CVD （Update model）

| Variables | Coefficient | HR | 95%CI | *P* |
| --- | --- | --- | --- | --- |
| Age group, yrs |  |  |  |  |
| 35~44 | Reference | 1.00 | - | *-* |
| 45~54 | 1.39 | 4.02 | 1.59-10.18 | <0.01 |
| 55~64 | 1.90 | 6.52 | 2.63-16.19 | <0.001 |
| 65~ | 2.50 | 12.13 | 4.59-30.67 | <0.001 |
| Male *vs.* Female | 0.37 | 1.44 | 1.09-1.91 | 0.01 |
| Current smoking | 0.32 | 1.37 | 1.04-1.81 | 0.03 |
| BMI | 0.03 | 1.04 | 1.00-1.07 | 0.07 |
| TIA | 0.87 | 2.38 | 1.50-3.78 | <0.001 |
| High LDL-C | 0.32 | 1.37 | 1.05-1.79 | 0.02 |
| Family of Hypertension | 0.21 | 1.24 | 0.92-1.67 | 0.16 |
| Family of stroke | 0.30 | 1.34 | 0.98-1.86 | 0.07 |
| Physical labor intensity |  |  |  |  |
| Light or moderate | Reference | 1.00 | - | *-* |
| Heavy | -0.39 | 0.67 | 0.49-0.92 | 0.01 |
| SBP grade (mm Hg) |  |  |  |  |
| < 140 | Reference | 1.00 | - | *-* |
| 140~159 | 0.10 | 1.11 | 0.60-2.03 | 0.75 |
| 160~179 | 0.32 | 1.37 | 0.74-2.55 | 0.32 |
| 180~199 | 0.69 | 1.99 | 1.02-3.86 | 0.04 |
| ≥ 200 | 0.98 | 2.67 | 1.27-5.56 | <0.01 |
| DBP grade (mm Hg) |  |  |  |  |
| < 90 | Reference | 1.00 | - | *-* |
| 90~109 | 0.26 | 1.29 | 0.96-1.74 | 0.09 |
| ≥110 | 0.89 | 2.43 | 1.42-4.16 | <0.01 |

BMI, body mass index; TIA, transient ischemic attack; LDL-C, low-density lipoprotein cholesterol ; SBP, systolic blood pressure; DBP, diastolic blood pressure.


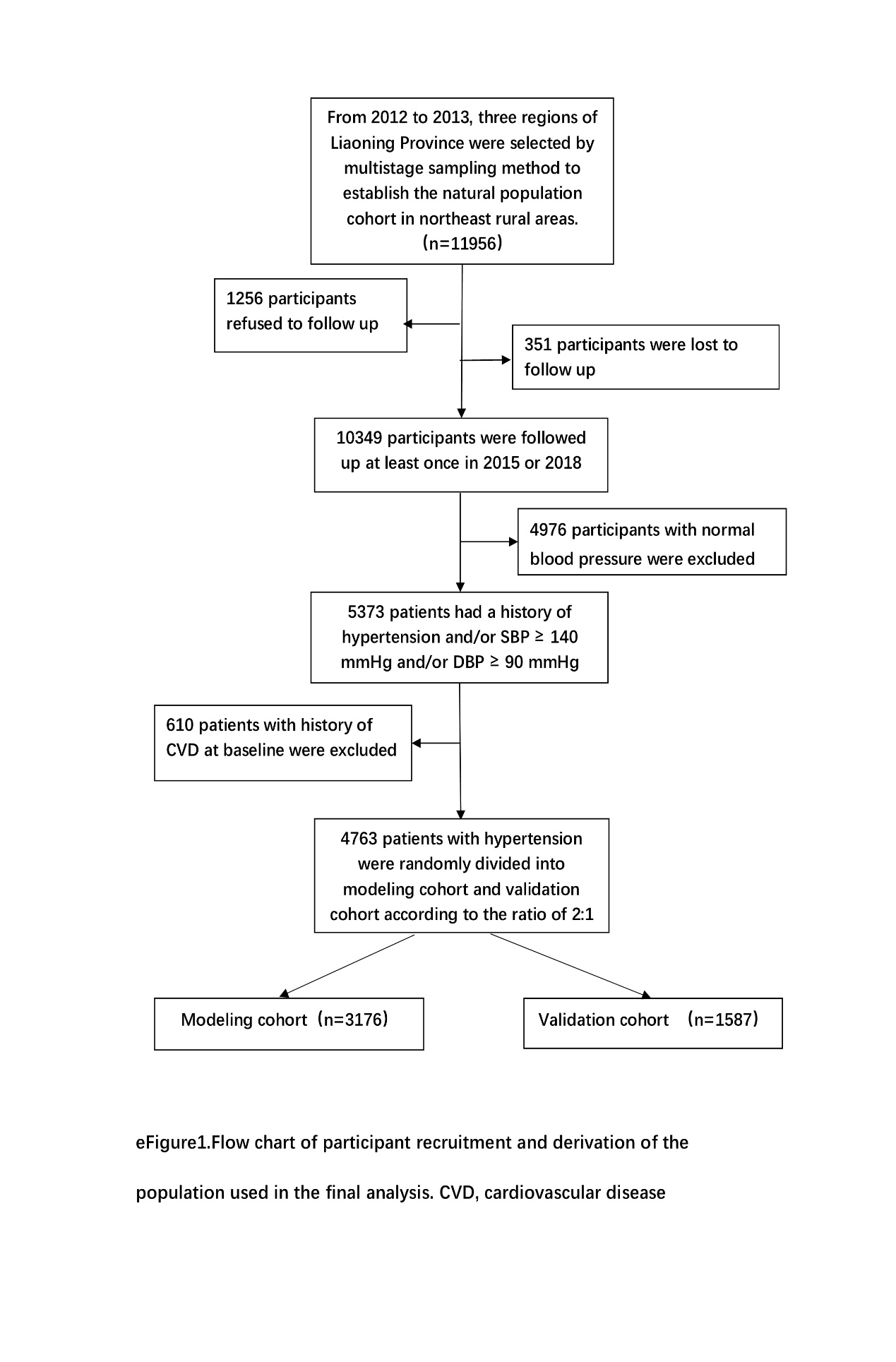


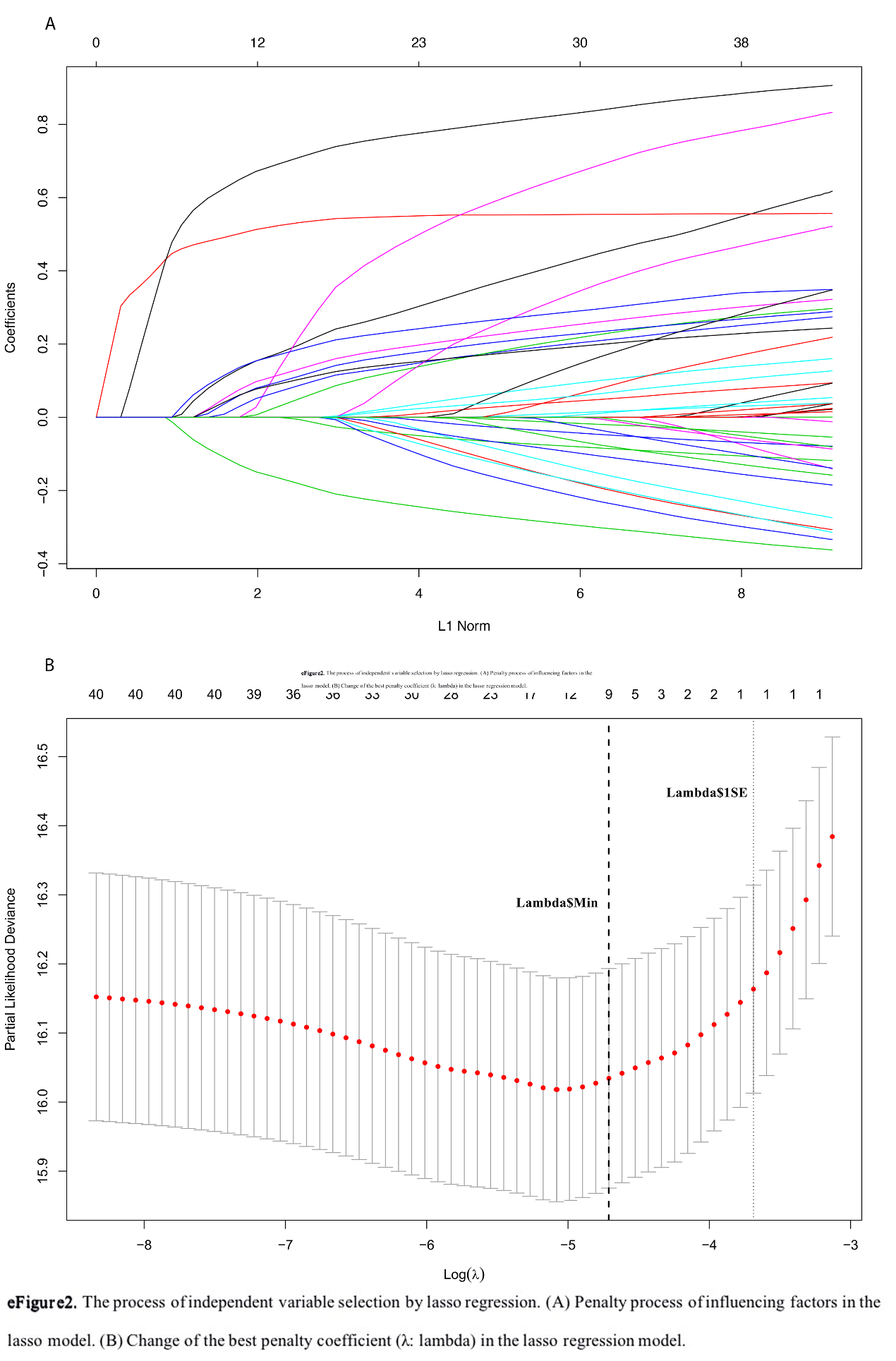


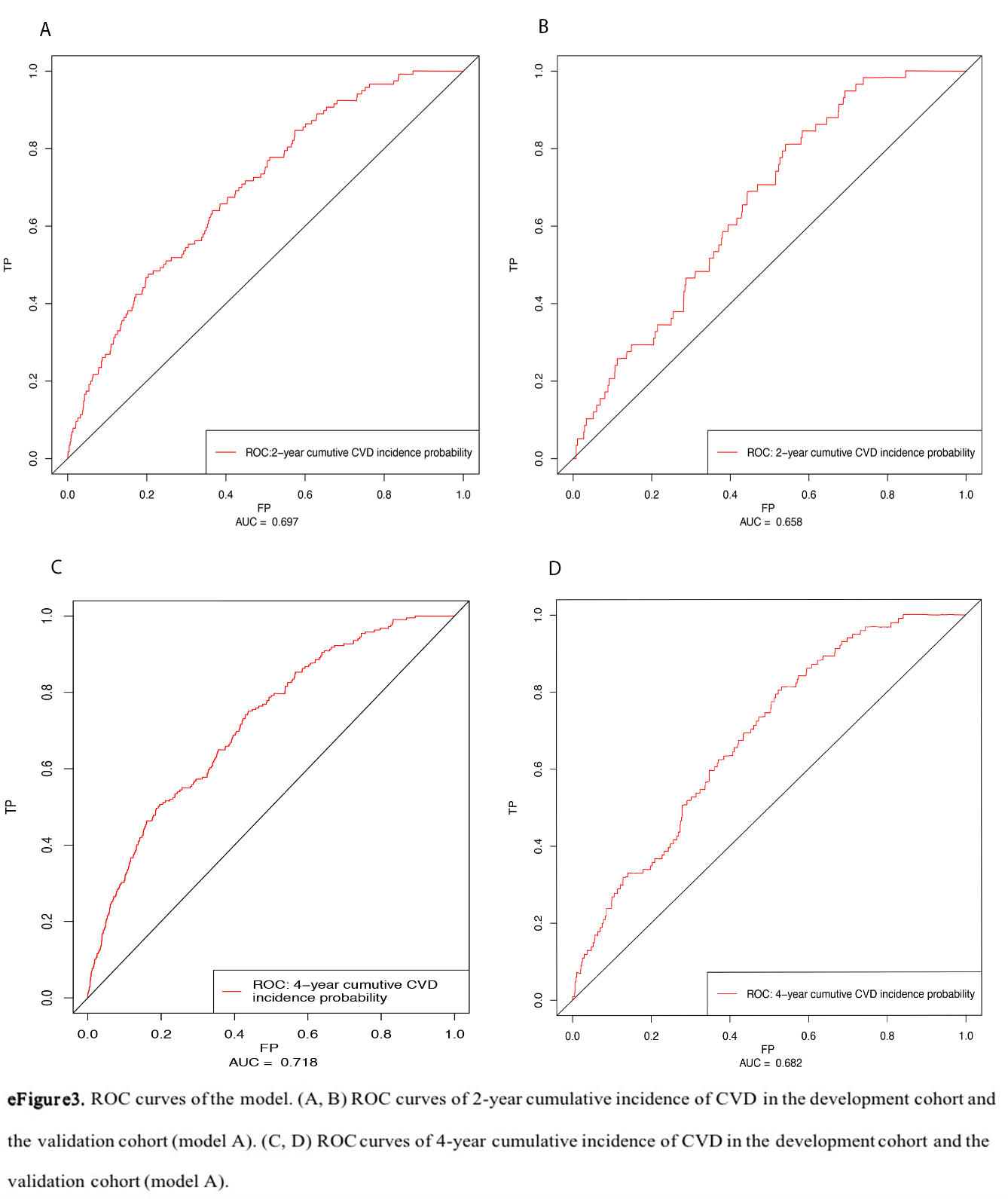


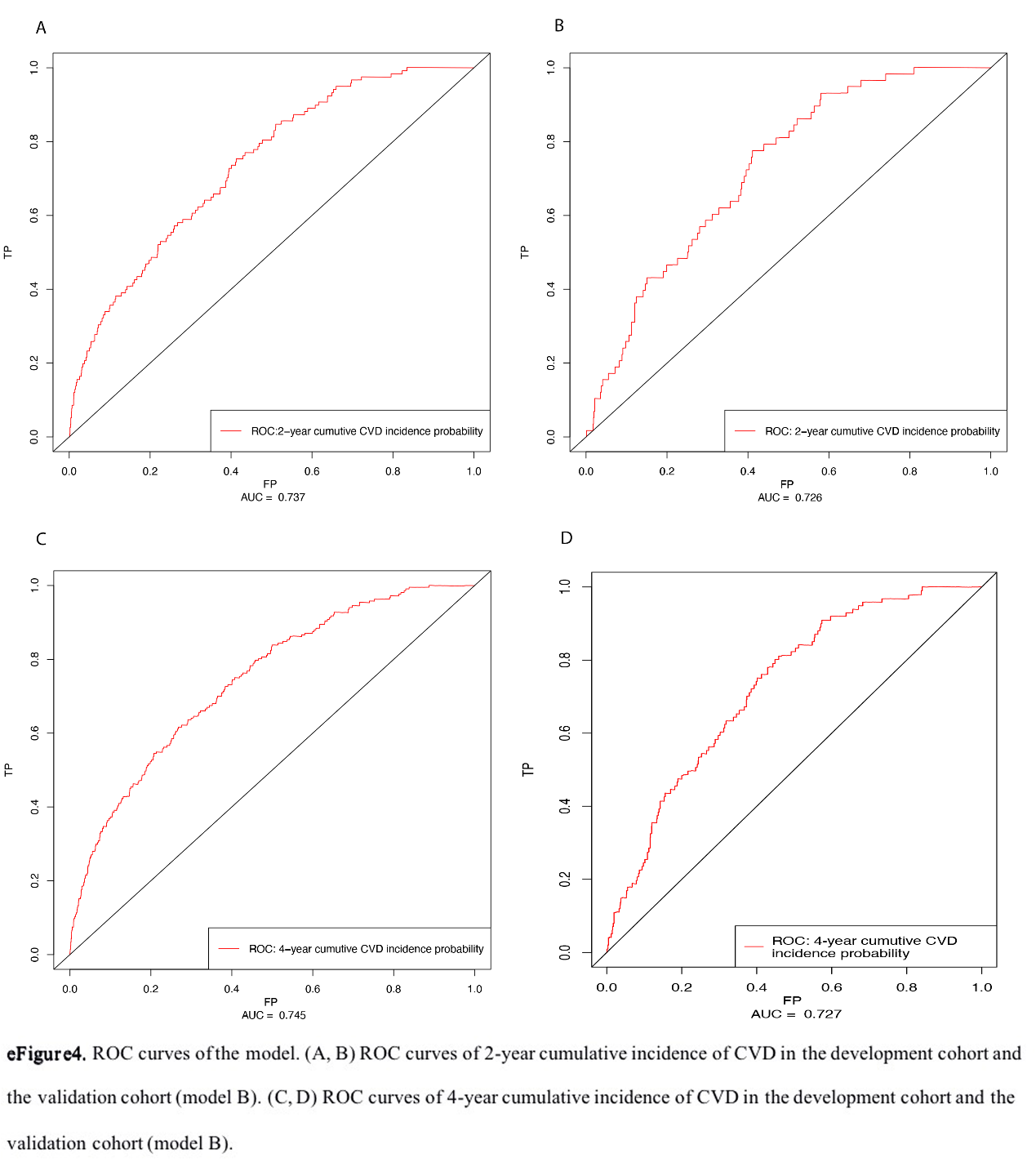

Supplement: Supplementary file 1 — Supporting material [file JCH-24-38-s001.docx]
